# Comparative Outcomes of Kirschner Wire Fixation Versus Locking Plate Osteosynthesis in Fifth Metacarpal Neck Fractures: A 20-Year Retrospective Analysis

**DOI:** 10.3390/medicina62091715

**Published:** 2026-09-07

**Authors:** Sung Huang Laurent Tsai, Shiny Chih-Hsuan Wu, Yung-Chuan Liu, Po-Ju Lai, Chun-Ying Cheng

**Affiliations:** 1 Department of Orthopedics, Taipei Medical University Hospital, Taipei 110, Taiwan; 2 Department of Orthopaedics, School of Medicine, College of Medicine, Taipei Medical University, Taipei 110, Taiwan; 3 Department of Biomedical Engineering, National Taiwan University, Taipei 106, Taiwan; 4 Graduate Institute of Clinical Medicine, College of Medicine, Taipei Medical University, Taipei 110, Taiwan; 5 Department of Orthopedics, Sports Section, Chang Gung Memorial Hospital, Linkou, Taoyuan 333, Taiwan; 6 Department of Orthopedic Surgery, Jen-Ai Hospital, Taichung 412, Taiwan; 7 Department of Nursing, College of Nursing, Hungkuang University, Taichung 433, Taiwan; 8 Division of Orthopedic Traumatology, Department of Orthopedic Surgery, Chang Gung Memorial Hospital, Taoyuan 333, Taiwan

**Keywords:** fifth metacarpal neck fracture, kirschner wire fixation, locking plate osteosynthesis, hand fracture fixation, metacarpal fracture outcomes, intramedullary fixation, radiographic union, return to work, functional hand recovery, comparative surgical techniques

## Abstract

*Background and Objectives:* Fifth metacarpal neck fractures are commonly encountered in hand surgery, yet optimal surgical fixation remains debated. This study compares Kirschner wire (K-wire) fixation and locking plate osteosynthesis in terms of radiological and functional outcomes. *Materials and Methods:* A retrospective cohort of 30 patients (mean age 45) from 2003–2022 was reviewed. Patients underwent either antegrade K-wire fixation or dorsal/volar locking plate fixation. Outcomes assessed included union time, angular and length deformity correction, return to work, and complication rates. *Results:* Both groups achieved similar final angulation correction (85%, *p* = 0.72). K-wire fixation achieved significantly faster radiographic union (K-wire: 3.5 months; plate: 4.5 months, *p* = 0. 019). K-wires allowed earlier intervention (6.0 (3.0–9.0) vs. 14.0 (9.0–22.0) days to surgery, *p* < 0.05) but had a 6.7% malunion rate. Plating allowed earlier return to work and avoided pin-tract issues. Functional scores (QuickDASH) and grip strength restoration (>90%) were statistically similar. *Conclusions:* Both K-wire fixation and locking plate osteosynthesis achieved bone union and satisfactory functional recovery in this retrospective cohort. However, given the small sample size and observational nature of the study, surgical selection should be tailored to individual patient presentation and surgeon preference until larger prospective trials are available.

## 1. Introduction

Fifth metacarpal neck fractures, commonly referred to as “boxer’s fractures,” account for approximately 10% of all hand fractures and are particularly prevalent in young adult males following axial trauma to the clenched fist [1]. While many of these fractures are amenable to conservative treatment, surgical intervention is often recommended when deformity exceeds accepted thresholds—typically more than 30–40° of dorsal angulation, 2–5 mm of shortening, or the presence of rotational malalignment which can significantly compromise hand function [2,3].

Two primary surgical options have emerged: percutaneous Kirschner wire (K-wire) fixation and locking plate osteosynthesis. K-wire fixation remains popular due to its simplicity, minimal invasiveness, and low cost. It allows preservation of soft tissue and periosteal blood supply, and is particularly useful for acute, minimally comminuted fractures. However, it typically requires postoperative immobilization and carries risks such as pin-site infection, wire migration, and limited control over rotational stability [4].

Locking plate osteosynthesis, in contrast, provides rigid internal fixation and has been advocated for comminuted or unstable fractures where precise reduction and early mobilization are prioritized [5]. Plates offer superior biomechanical stability in axial and torsional loading models, which is especially beneficial in polytraumatized or high-demand patients [6]. However, they also carry increased risks, including hardware prominence, extensor tendon irritation, and greater surgical dissection, and may require hardware removal if symptomatic [7].

Despite these differences in approach and biomechanics, clinical studies have reported similar long-term functional outcomes, with little consensus on superiority. QuickDASH scores, grip strength recovery, and range of motion often converge between the two techniques over time, suggesting that patient-specific factors such as fracture pattern, occupation, and surgical timing may be more critical in determining optimal treatment [8,9].

This retrospective study aims to evaluate the comparative outcomes of K-wire fixation versus locking plate osteosynthesis in the management of fifth metacarpal neck fractures over a 20-year period. By analyzing union rates, radiographic correction, return to function, and complications, we seek to clarify the indications for each technique and provide practical guidance on their selection in clinical practice.

## 2. Material and Methods

This was a retrospective cohort study conducted over a 20-year period (2003–2022) at a single tertiary hand surgery center. The objective was to compare clinical and radiographic outcomes in patients with isolated fifth metacarpal neck fractures treated either with Kirschner wire (K-wire) fixation or locking plate osteosynthesis. Ethical approval was obtained from the institutional review board, and informed consent was waived due to the retrospective nature of the analysis.

Over the 20-year period (2003–2022), 248 consecutive adult patients with closed fifth metacarpal neck fractures were treated at our department. Nonoperative management was successful in 212 patients (85.5%). Thirty-six patients underwent surgical fixation based on institutional threshold criteria (>30° dorsal angulation, >3 mm shortening, or rotational malalignment). Six patients were excluded (2 open fractures, 1 associated carpal injury, 1 pathological fracture, and 2 lost to follow-up before 12 months). The final analyzed cohort comprises 30 consecutive patients (15 antegrade K-wire, 15 locking plate) with 0% missing outcome data at final follow-up.

Eligible patients were adults aged 18 years or older with closed, isolated fifth metacarpal neck fractures exhibiting greater than 30 degrees of dorsal angulation or more than 3 mm of shortening on initial radiographs. Exclusion criteria included polytrauma, open fractures, injuries extending into the metacarpal base or shaft, pathological fractures, metabolic bone disease, or any form of immunosuppression. All procedures were performed by a single experienced hand surgeon under regional anesthesia with intraoperative fluoroscopic guidance. In the K-wire group, closed reduction was achieved using the Jahss maneuver, followed by antegrade insertion of two pre-bent 1.4 mm Kirschner wires through a small dorsal incision at the metacarpal base. The wires were placed in a divergent intramedullary configuration to enhance rotational and angular stability. A short-arm ulnar gutter splint was applied for three weeks postoperatively, with wire removal scheduled between the third and fourth week based on radiographic healing. For the locking plate group, 3/15 patients (86.7%) were treated via a dorsal longitudinal approach and 2/15 (13.3%) via a dorso-ulnar approach to apply a five-hole titanium locking plate with bicortical screw fixation. Direct or indirect fracture reduction was achieved prior to fixation, and confirmed fluoroscopically. Postoperatively, both cohorts were initially placed in a short-arm ulnar gutter splint for three weeks. However, the post-splinting progression differed between groups based on fixation biomechanics. In the locking plate group, the initial 3-week splint served purely for soft-tissue resting and surgical wound healing. Upon splint removal at week 3, the absolute stability provided by bicortical screw fixation permitted immediate active and passive range of motion, forceful grasping, and progressive mechanical loading without external protection. In the antegrade K-wire group, the splint provided essential fracture stability. Following splint removal and wire extraction between the third and fourth postoperative weeks, gentle active motion was initiated, but forceful loading, heavy manual labor, and resistive grasping were restricted until solid bridging callus was confirmed on orthogonal radiographs (typically 6–8 weeks postoperatively) to prevent pin loosening or secondary loss of reduction. Hardware was retained unless it became symptomatic. Outcome measures included radiographic and functional assessments. Radiographic parameters such as dorsal angulation and metacarpal shortening were measured using 30-degree pronated oblique views. Time to union was defined radiographically as evidence of bridging callus on at least three cortices in orthogonal views. Functional outcomes included grip strength using a Jamar dynamometer (Performance Health, Downers Grove, IL, USA), metacarpophalangeal (MCP) joint range of motion compared to the contralateral side, and QuickDASH scores for upper extremity disability. Additional data collected included time from injury to surgery, time to return to work (RTW), and complications such as malunion, pin migration, infection, or hardware irritation. All data were analyzed using STATA version 16.0 (StataCorp LLC, College Station, TX, USA). Continuous variables were evaluated for distributional normality using the Shapiro–Wilk test. Normally distributed variables were expressed as mean ± standard deviation (SD) and compared using the independent-samples Student’s t-test. Non-normally distributed variables (such as time from injury to surgery) were presented as median (interquartile range, IQR) and evaluated using the Mann–Whitney U test. Categorical variables were reported as frequencies (percentages) and evaluated with Fisher’s exact test or the Chi-square test. A two-tailed *p*-value of <0.05 was defined as statistically significant.

## 3. Result

### 3.1. Demographics and Baseline Characteristics

A total of 30 patients were included in the final analysis, with 15 patients in the K-wire fixation group and 15 in the locking plate group (Table 1). The mean age across both groups was 45 years, and 86.7% (n = 26) were male. The two groups had no significant difference regarding age, sex, hand dominance (K-wire: 60% vs. plate: 53.3%, *p* = 0.77), or smoking status (K-wire: 53.3% vs. plate: 60%, *p* = 0.33).

### 3.2. Time to Surgery

The median time from injury to surgery was significantly shorter in the K-wire group (6.0 (3.0–9.0) days) compared to the locking plate group (14.0 (9.0–22.0) days), reflecting the preference for plating in delayed or unstable fracture presentations (*p* < 0.05) (Table 1).

#### 3.2.1. Radiographic Outcomes

**Figure 1:**
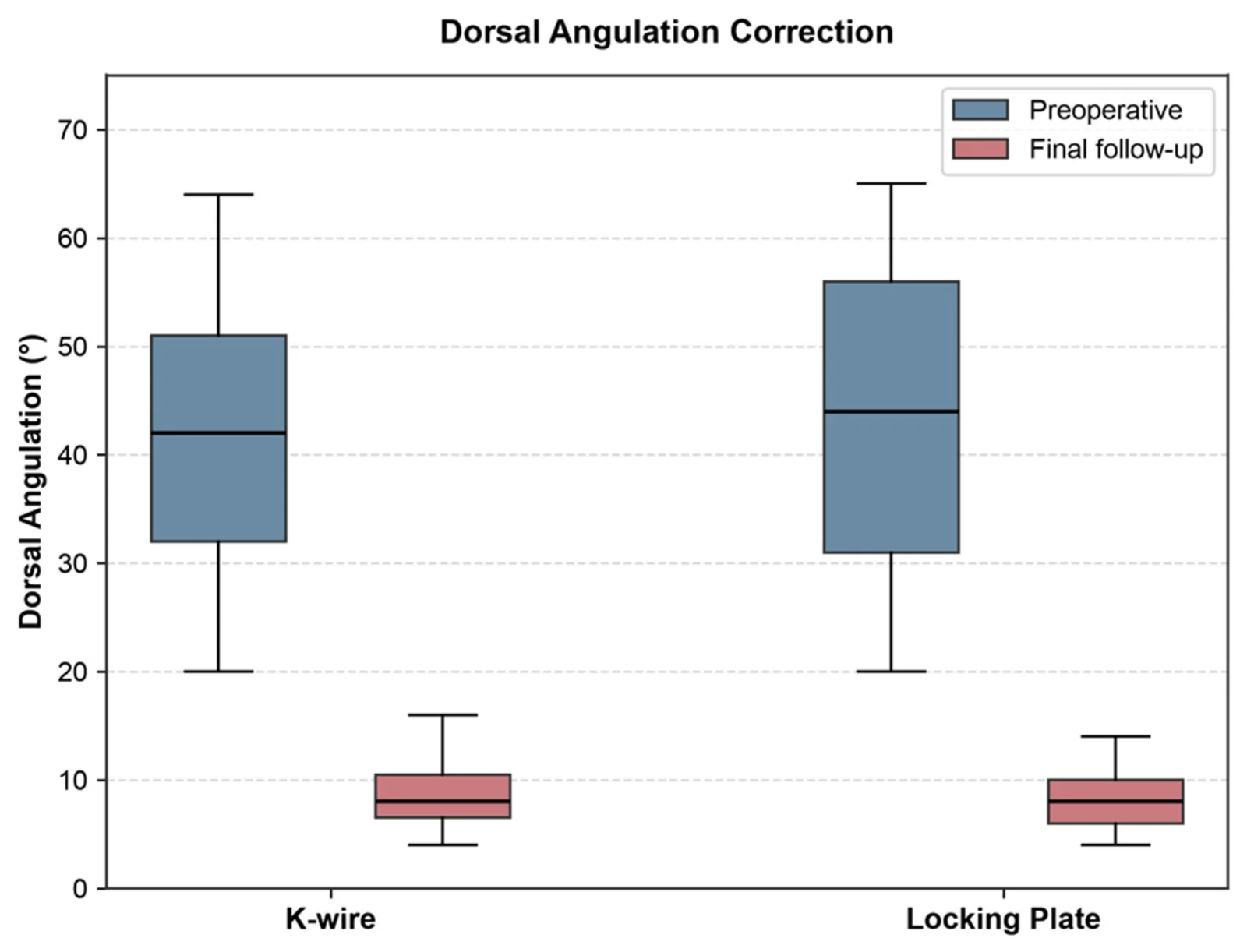
Box-and-whisker plot comparing preoperative and final follow-up dorsal angulation (°) in fifth metacarpal neck fractures treated with Kirschner wire versus locking plate fixation. The horizontal line inside each box denotes the median, the box boundaries represent the interquartile range (IQR), and the whiskers indicate the range.

At final follow-up, both groups demonstrated effective correction of dorsal angulation. The mean preoperative angulation was comparable between the groups and was reduced postoperatively by an average of 85% in both cohorts (*p* = 0.72). The mean residual dorsal angulation at follow-up was 8.2° in the K-wire group and 7.9° in the plating group (Figure 1).

**Figure 2:**
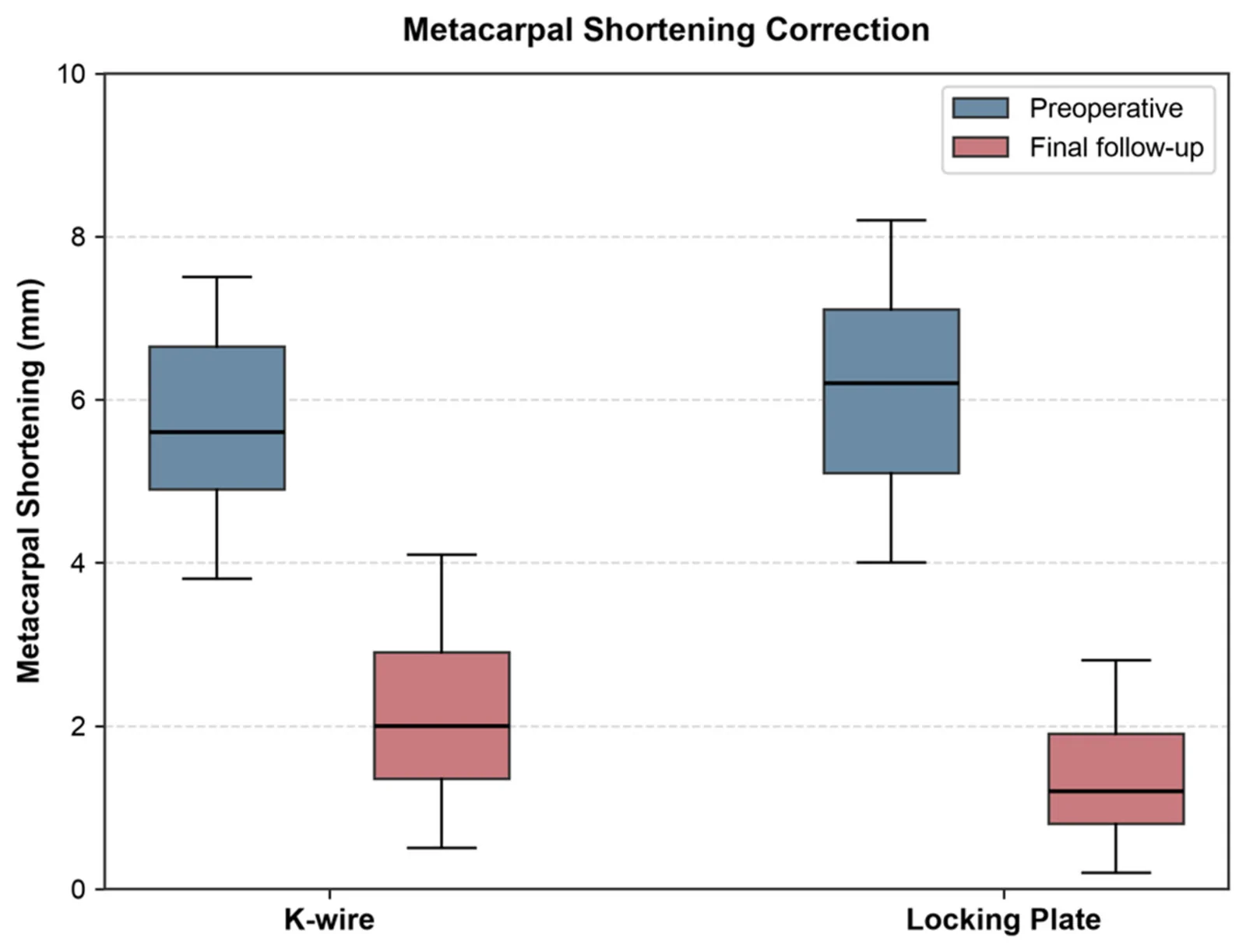
Box-and-whisker plot illustrating preoperative and postoperative fifth metacarpal shortening (mm) between the K-wire and locking plate groups. While both techniques achieved reduction, plating resulted in significantly less residual shortening. (*p* = 0.028).

With regard to metacarpal shortening, both groups achieved acceptable anatomical alignment. The average postoperative shortening was 2.1 mm in the K-wire group and 1.3 mm in the plating group, a difference that reached statistical significance (*p* = 0.028) (Figure 2).

**Figure 3:**
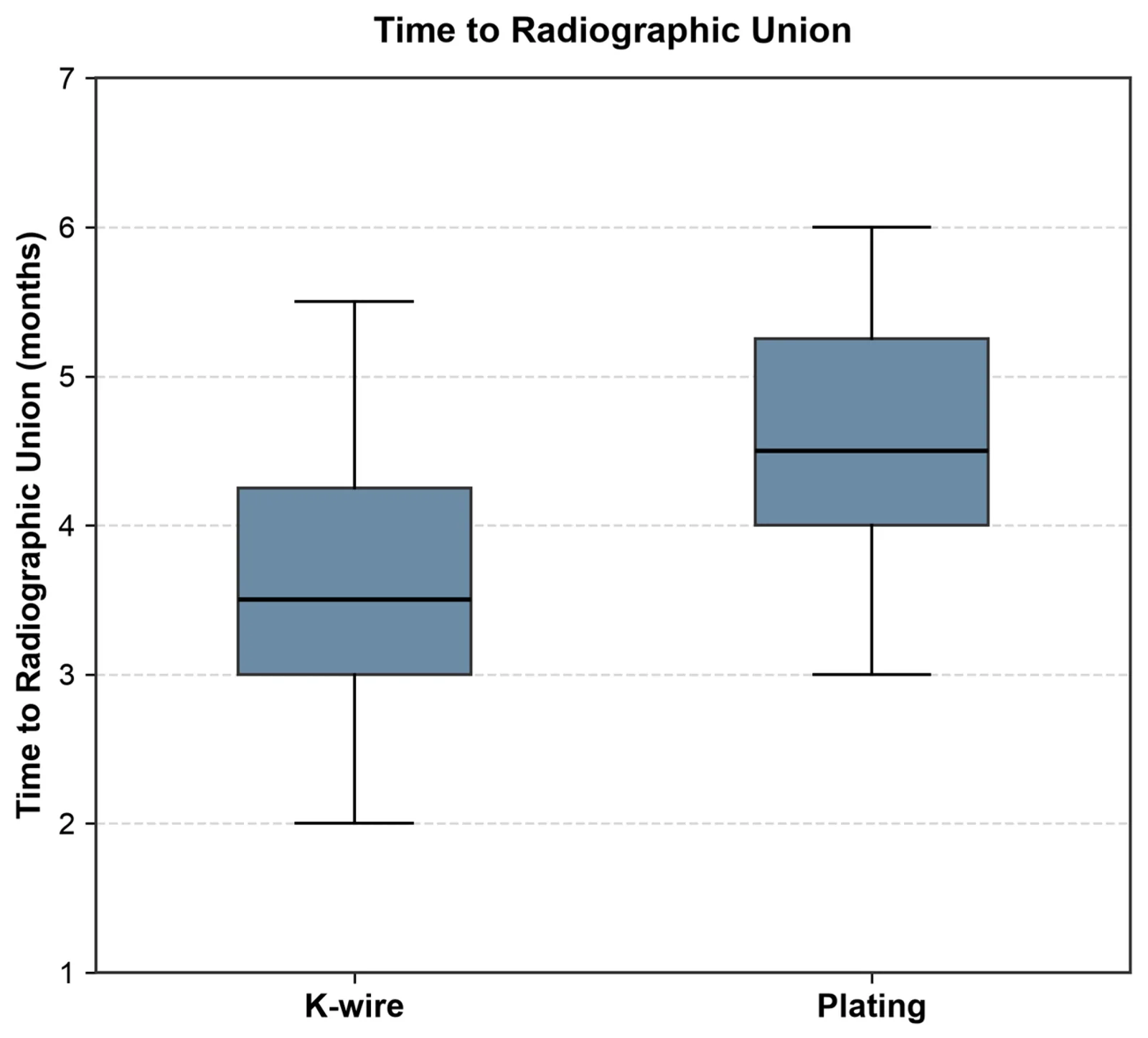
Time to radiographic union (months) for fifth metacarpal neck fractures treated with Kirschner wire versus locking plate fixation. K-wire fixation achieved significantly faster radiographic union [*p* = 0.019].

Time to radiographic union averaged 3.5 months in the K-wire group and 4.5 months in the plate group (*p* = 0.019), with all fractures uniting by final follow-up (Figure 3). No cases of delayed or nonunion were observed.

#### 3.2.2. Functional Outcomes

QuickDASH scores at final follow-up were low in both groups, indicating excellent subjective function. The K-wire group had a mean score of 8.2, while the plate group scored 7.9 (*p* = 0.89), demonstrating no significant difference in patient-reported outcomes. Grip strength recovery, as measured with a Jamar dynamometer, exceeded 90% of the contralateral hand in both groups. Similarly, the range of motion at the metacarpophalangeal (MCP) joint was well-preserved, with the K-wire group averaging 91.25% of contralateral MCP motion and the plating group 94% (*p* = 0.167).

#### 3.2.3. Return to Work and Activity

All 30 patients were actively employed prior to injury (eligible denominator = 15 per group). Time to return to work (RTW) was defined as the duration in weeks from the date of index surgery to full, unrestricted occupational resumption without physical modification, ascertained through documented clinical and occupational health follow-up records.

**Figure 4:**
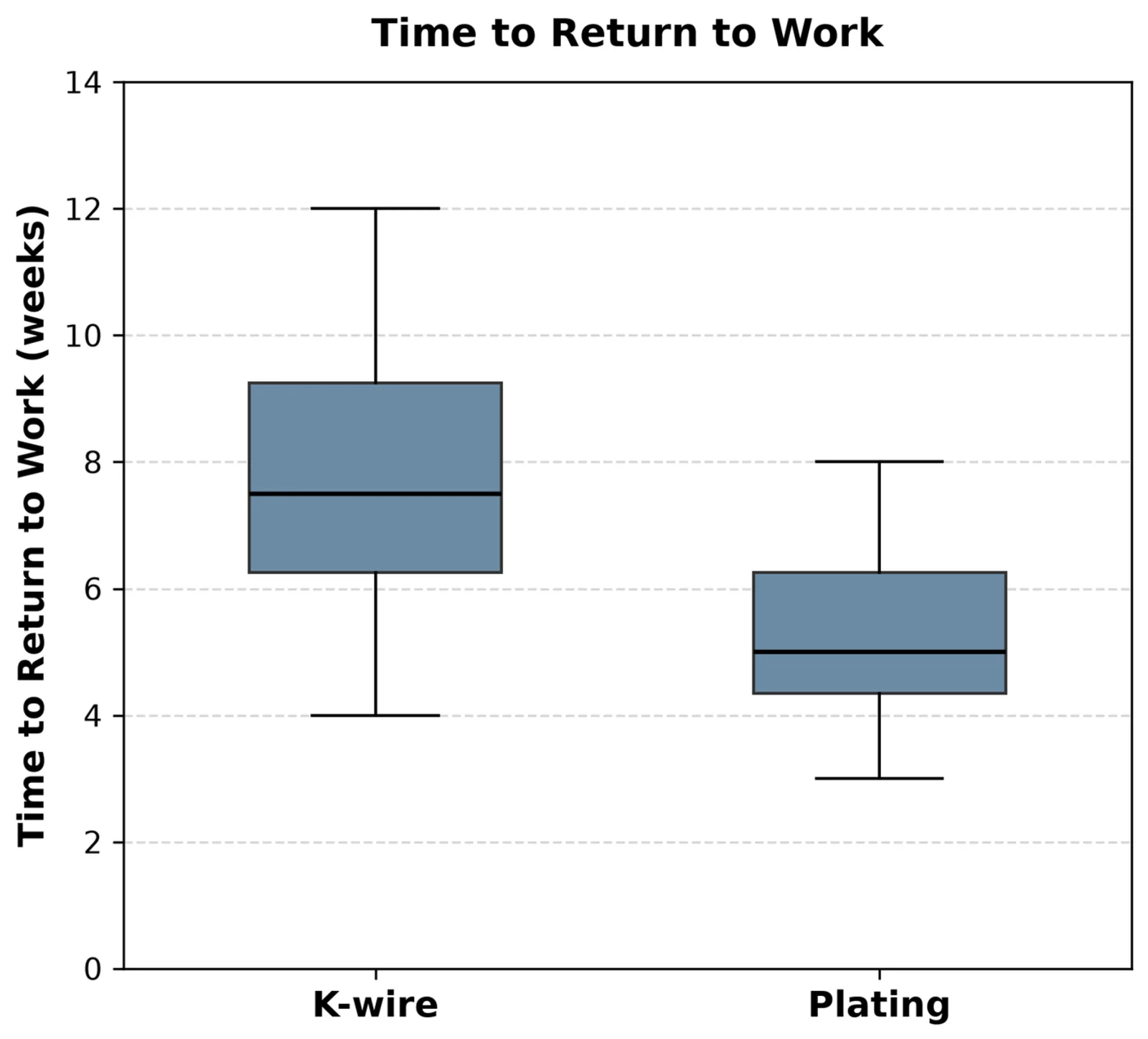
Time to unrestricted return to work (weeks) following surgical fixation. Patients in the locking plate group returned to occupational duties significantly earlier than those in the K-wire group (5.2 ± 1.5 vs. 7.6 ± 2.26 weeks, *p* = 0.002).

Return to work or play was significantly faster in the plating group, with a mean of 5.2 ± 1.5 weeks compared to 7.6 ± 2.4 weeks in the K-wire group (*p* = 0.002) (Figure 4). This earlier occupational clearance and functional loading in the plating group was facilitated by the rigid internal construct, which permitted immediate, confident hand loading as soon as the 3-week protective resting splint was discontinued, whereas the K-wire group required continued load restrictions until complete radiographic consolidation.

#### 3.2.4. Complications

In the K-wire group, two patients experienced pin migration, and one patient (6.7%) developed a clinically significant malunion with residual angulation >15°, though this did not require reoperation. No infections, neurovascular injuries, or hardware failures were observed. In the plating group, one patient developed extensor tendon irritation necessitating elective hardware removal. There were no cases of infection, hardware loosening, or tendon rupture (Table 2).

## 4. Discussion

The optimal surgical treatment for fifth metacarpal neck fractures remains a topic of ongoing debate. Our findings reinforce that both Kirschner wire (K-wire) fixation and locking plate osteosynthesis offer reliable outcomes in terms of radiological union, deformity correction, and functional recovery. However, the growing body of recent literature suggests nuanced advantages and limitations that should guide surgical choice based on patient-specific factors.

In our cohort, both fixation methods achieved satisfactory correction of dorsal angulation and metacarpal shortening, echoing results from multiple studies. Wang et al. observed comparable radiographic and functional outcomes in an adolescent cohort with fifth metacarpal neck fractures treated with either percutaneous K-wire fixation or elastic stable intramedullary nailing, noting that both methods restored alignment with low complication rates [10]. Scale et al. further confirmed that single intramedullary K-wire fixation yields high union rates and excellent range of motion in displaced fractures, especially when postoperative immobilization is limited [11].

Locking plate fixation offers increased construct rigidity, making it particularly suitable for comminuted fractures or when early mobilization is necessary. Although both patient cohorts underwent a standard 3-week period of postoperative ulnar gutter splinting for initial soft-tissue protection, their subsequent rehabilitation timelines diverged. Locking plate osteosynthesis establishes rigid bicortical stability, thereby enabling earlier functional loading and unrestricted occupational clearance immediately following splint removal at 3 weeks. Conversely, while antegrade intramedullary K-wire fixation preserves the periosteal biology and minimizes soft-tissue dissection, the construct relies on relative stability and requires transfixing hardware retention. Consequently, full mechanical loading and return to heavy occupational duties in the pinning group had to be deferred until solid radiographic bridging occurred, explaining the observed difference in return-to-work timelines 5.2 vs. 7.6 weeks. We explicitly contextualize that the observed faster RTW in the plating group was protocol-dependent. Plate fixation provided rigid internal construct stability, allowing immediate active loading and occupational clearance upon removal of the 3-week resting splint. Conversely, the K-wire group required continued external pin protection until wire extraction (weeks 3–4) and loading restrictions until bridging callus formation (~6–8 weeks) to prevent loss of reduction. Biomechanical investigations have further elucidated the mechanical profiles of different constructs. Wallace et al. compared intramedullary threaded nails with dorsal locking plate constructs, showing that intramedullary implants can provide robust construct stiffness while avoiding extensive periosteal stripping [12]. Chiu et al. also noted that, while cosmetically preferable, buried K-wires might underperform mechanically compared to locking plate constructs in phalangeal and metacarpal fractures [13].

Our study supports recent findings from Chiu et al., who developed a hybrid K-wire and cerclage wire construct for transverse metacarpal neck fractures that approached the biomechanical strength of plate fixation while preserving soft tissue integrity [13]. Similarly, Zhu et al. showed that combining a locking plate with crossed K-wires produced superior radiographic alignment and functional range of motion compared to plates alone [7].

Complication profiles varied between groups, as reflected in both our data and current literature. K-wire fixation carries a known risk of pin tract infection, migration, and secondary displacement, especially if early mobilization is not cautiously managed. McMahon and Ibrahim found that retrograde K-wire placement, while technically simpler, is associated with higher risk of extensor tendon irritation unless wires are properly buried or bent subcutaneously [14]. In contrast, plate fixation often avoids external fixation-related issues but may require implant removal due to dorsal hardware prominence, as shown by Nelson et al. in a large outcomes study comparing pin versus plate fixation in metacarpal shaft fractures [15].

Functional recovery in our study was comparable between groups. This aligns with recent outcomes reported by Lee et al., who compared percutaneous pinning and open locking plating for intra-articular fifth metacarpal base fractures, finding no significant difference in grip strength or range of motion at final follow-up [16]. Likewise, Pasquino et al. reported similar patient satisfaction and quality of life between locking plates and intramedullary K-wires in midshaft fractures [17].

Finally, new trends suggest increasing acceptance of antegrade elastic nailing, especially in adolescents. Fan et al. found this approach preserved epiphyseal growth while achieving high union and functional success rates [18], indicating growing flexibility in fixation strategies across age groups.

Several limitations must be acknowledged. First, the small sample size (n = 30) accrued over a 20-year study duration limits statistical power, increasing the risk of type II errors for functional outcomes and complication rates. Second, the non-randomized, retrospective design introduces treatment-selection bias. Third, over the 20-year study period, subtle evolutions in low-profile locking plate profiles, screw locking mechanisms, and accelerated hand rehabilitation protocols may have occurred, representing historical confounders. Thus, our results reflect longitudinal institutional observations rather than a controlled equivalence trial.

Another limitation is the absence of standardized, prospective quantitative assessments of rotational malalignment (such as digital cascade overlap angles during active fist closure). Although intraoperative tenodesis and clinical records confirmed the absence of gross scissoring at final follow-up, subtle rotational malalignment may not have been fully captured.

## 5. Conclusions

In this 20-year retrospective series of surgically treated fifth metacarpal neck fractures, both K-wire fixation and locking plate osteosynthesis achieved radiographic union and satisfactory functional recovery with low overall complication rates. Fixation choice in clinical practice was heavily influenced by time from injury to surgery. While locked plating was associated with earlier return to work, it entailed open exposure and potential hardware irritation. Given the observational design and small sample size, surgical decision-making should be individualized based on fracture pattern, timing, and surgeon familiarity until larger prospective studies are conducted.

## Figures and Tables

**Table 1:** Baseline and Surgical Characteristics.

Variable	K-Wire (n = 15)	Plating (n = 15)	Difference (95% CI)	*p*-Value
Age, mean (SD), years	45 (12.5)	45 (12.9)	0.0 (−9.49 to 9.49)	0.999
Male sex, n (%)	13 (86.7)	13 (86.7)	—	1.000
Dominant hand, n (%)	9 (60.0)	8 (53.3)	—	0.713
Time to surgery, days, median (IQR)	6 (3–9)	14 (9–22)	Mann–Whitney U = 43.5	0.008 *
Follow-up duration (months)	14.2±4.8	15.6±5.1	−1.4 (−5.08 to 2.28)	0.445
Residual dorsal angulation (degree)	8.2±2.5	7.9±2.6	0.33 (−1.57 to 2.24)	0.724
Postoperative shortening (mm)	2.1±1.1	1.3±0.8	0.8 (−0.09 to 1.51)	0.028 *
Time to union, months	3.5±1.0	4.5±1.1	−0.93 (−1.70 to −0.17)	0.019 *
Grip strength (% of uninjured)	91.5±6.2	93.8±5.7	−2.33 (−6.78 to 2.11)	0.291
MCP active ROM (degree)	82.5±6.8	85.1±6.2	−2.53 (−7.42 to 2.35)	0.294
QuickDASH score	8.2±4.1	7.9±3.9	0.33 (−2.66 to 3.33)	0.821
Return to work (weeks)	7.6±2.3	5.2±1.5	2.4 (0.98 to 3.82)	0.002 *

Data are presented as mean ± standard deviation (SD) for normally distributed continuous variables, median (interquartile range, IQR) for skewed continuous variables, or frequency (percentage, %) for categorical variables. Between-group comparisons were evaluated using the two-tailed independent-samples Student’s *t*-test, Mann-Whitney *U* test, or Fisher’s exact test as appropriate. * *p* < 0.05, indicating a statistically significant difference between the K-wire and locking plate groups. Abbreviations: CI, confidence interval; IQR, interquartile range; K-wire, Kirschner wire; MCP, metacarpophalangeal; ROM, range of motion; SD, standard deviation.

**Table 2:** Summary of Postoperative Complications and Interventions.

Complication	K-Wire (n = 15)	Plating (n = 15)	*p*-Value	Clinical Course/Management
Overall Complication Rate, n (%)	3 (20.0%)	1 (6.7%)	0.598	—
Pin migration (requiring early wire removal)	2 (13.3%)	0(0.0%)	0.483	Managed by outpatient wire removal at week 3 without loss of alignment.
Symptomatic malunion (>15° angulation)	1 (6.7%)	0 (0.0%)	1.000	Residual dorsal angulation of 18°; managed nonoperatively with satisfactory grip strength (>85%).
Extensor tendon irritation (requiring plate removal)	0 (0.0%)	1 (6.7%)	1.000	Underwent elective hardware removal at 9 months postoperatively with symptom resolution.
Superficial/deep surgical site infection	0 (0.0%)	0 (0.0%)	—	None observed.
Complex regional pain syndrome (CRPS)	0 (0.0%)	0 (0.0%)	—	None observed.

## Data Availability

The data presented in this study are available from the corresponding author upon reasonable request. The data are not publicly available because they contain information that could compromise patient privacy.
